# Changes in expressions of genes involved in the regulation of cellular processes in mucopolysaccharidoses as assessed by fibroblast culture-based transcriptomic analyses

**DOI:** 10.1007/s11011-020-00614-2

**Published:** 2020-09-04

**Authors:** Lidia Gaffke, Karolina Pierzynowska, Karolina Krzelowska, Ewa Piotrowska, Grzegorz Węgrzyn

**Affiliations:** Department of Molecular Biology, University of Gdańsk, Wita Stwosza 59, 80-308 Gdańsk, Poland

**Keywords:** Mucopolysaccharidoses, Transcriptomics, Regulation of cellular processes

## Abstract

Recent studies indicated that apart from lysosomal storage of glycosaminoglycans (GAGs), secondary and tertiary changes in cellular processes may significantly contribute to development of disorders and symptoms occurring in mucopolysaccharidoses (MPS), a group of lysosomal storage diseases in which neurodegeneration is specific for most types and subtypes. In this report, using transcriptomic data, we demonstrate that regulation of hundreds of genes coding for proteins involved in regulations of various cellular processes is changed in cells derived from patients suffering from all types and subtypes of MPS. Among such genes there are 10 which expression is significantly changed in 9 or more (out of 11) MPS types/subtypes; they include *IER3IP1, SAR1A, TMEM38B, PLCB4, SIN3B, ABHD5, SH3BP5, CAPG, PCOLCE2,* and *MN1*. Moreover, there are several genes whose expression is changed over log_2_ > 4 times in some MPS types relative to control cells. The above analysis indicates that significant changes in expression of genes coding for various regulators of cellular processes may considerably contribute to development of cellular dysfunctions, and further appearance of specific symptoms of MPS, including neurodegeneration.

## Introduction

Mucopolysaccharidoses (MPS) are a group of inherited metabolic diseases (Zhou et al. 2020). They belong to lysosomal storage disorders (LSD) (Sun 2018) since the primary cause of them is a lack or significant decrease in activity of one of enzymes involved in degradation of glycosaminoglycans (GAGs). Due to the presence of specific mutations and enzymatic dysfunctions, undegraded GAGs accumulate in lysosomes and cause defects in cellular functions. There are 11 types and subtypes of MPS, depending on the kind of stored GAG(s) and enzymatic defect (Kubaski et al. 2020). In 7 out of 11 types/subtypes, central nervous system (CNS) is involved, due to severe neurodegenerative processes occurring in the course of the diseases (Kobayashi 2019). In fact, all MPS types are severe diseases with progressing symptoms appearing in virtually all tissues and organs (especially in neuronopathic forms). The average life span is estimated to about two decades (Kobayashi 2019; Zhou et al. 2020).

Initially, GAG storage was considered as the only cause of the disease (Dorfman and Matalon 1976; Kelly 1976). However, subsequent studies indicated that secondary and tertiary changes in cells might significantly contribute to development of the disease symptoms (Gaffke et al. 2019; Fecarotta et al. 2020). Very recent transcriptomic analyses indicated that there are hundreds of genes whose transcription is down- or up-regulated in MPS cells relative to control cells (Gaffke et al. 2020). These studies were based on the use of lines of fibroblasts derived from patients suffering from all types and subtypes of MPS. Despite obvious limitations of such experiments, like the use of cell types which cannot represent most of tissues in patients’ bodies, and representation of each MPS type/subtype by only one cell lines, the advantages of these studies were possibility to compare transcriptomic changes in all MPS types/subtypes in one experiment and under the same conditions, and possibility to identify genes whose expression is significantly changed in most types/subtypes. In such a way, it was possible to preliminarily identify the genes whose changed expression in MPS cells might be responsible for or contribute to disorders in various cellular processes, including apoptosis (Brokowska et al. 2020) and cell activation (Rintz et al. 2020), or even to disturbance in behavioral disorders (Pierzynowska et al. 2020). This has encouraged us to ask what genes coding for proteins involved in regulation of cellular processes reveal changed expression in MPS cells relative to controls. Therefore, results of relevant transcriptomic analyses are presented in this report.

## Materials and methods

Analyses performed in this report were based on the use of the RNA-seq data, deposited in the NCBI Sequence Read Archive (SRA), under accession number PRJNA562649 (Gaffke et al. 2020). The data were obtained on the basis of four biological repeats (understood as four independent experiments with one cell line at different passages, between 4th and 15th) of the experiment with fibroblasts derived from a healthy person and patients suffering from all known types and subtypes of MPS (Table 1). As described previously (Gaffke et al. 2020), Illumina TruSeq Stranded mRNA Library Prep Kit was used to prepare the mRNA libraries. Then, the cDNA libraries were sequenced employing a HiSeq4000 (Illumina, San Diego, CA, USA). Following parameters were used: PE150 (150 bp paired-end) and minimum 40 × 10^6^ of raw reads. This gave a minimum of 12 Gb of raw data per each sample. FastQC version v0.11.7 was used for quality assessment. Raw readings were mapped to the GRCh38 human reference genome from the Ensembl database. Hisat2 v. 2.1.0 program was used for this procedure. Cuffquant and Cuffmerge programs in version 2.2.1 and the GTF Homo_sapiens.GRCh38.94.gtf file from the Ensembl database were used to calculate the expression levels. The Cuffmerge program was started with the library-norm-method classic-fpkm parameter normalizing the expression values by means of the FPKM algorithm. Statistical significance was analyzed using one-way analysis of variance (ANOVA) on log_2_(1 + x) values which have normal continuous distribution. The false discovery rate (FDR) was estimated using the Benjamini–Hochberg method. For comparisons between two groups, post hoc Student’s t test with Bonferroni correction was employed. R software v3.4.3 was employed to conduct all statistical analyses. Transcript annotation and classification was performed using the BioMart interface for the Ensembl gene database.

**Table 1:** MPS types and characteristics of fibroblast lines derived from MPS patients and control cell line used in transcriptomic analyses

MPS type	Primary stored GAG(s)^a^	Mutated gene and its locus	Mutation(s) in the used fibroblast line^b^	Catalog number of the cell line^c^
MPS I	DS, HS	*IDUA*, 4p16.3	p.Trp402Ter/p.Trp402Ter	GM00798
MPS II	DS, HS	*IDS*, Xp28	p.His70ProfsTer29	GM13203
MPS IIIA	HS	*SGSH*, 17q25.3	p.Glu447Lys/p.Arg245His	GM00879
MPS IIIB	HS	*NAGLU*, 17q21	p.Arg626Ter/p.Arg626Ter	GM00156
MPS IIIC	HS	*HGSNAT*, 8p11.1	Not determined	GM05157
MPS IIID	HS	*GNS*, 12q14	p.Arg355Ter/p.Arg355Ter	GM05093
MPS IVA	KS, CS	*GALNS*, 16q24.3	Not determined	GM00593
MPS IVB	KS, CS	*GLB1*, 3p22.3	p.Trp273Leu/p.Trp509Cys	GM03251
MPS VI	DS	*ARSB*, 4q14.1	Not determined	GM03722
MPS VII	DS, HS, CS	*GUSB*, 7q21.11	p.Trp627Cys/p.Arg356Ter	GM00121
MPS IX	HA	*HYAL1*, 3p.21.3	Not determined	GM17494
Control line (HDFa)	None	N/A	N/A	N/A

^a^*Abbreviations*: *CS* chondroitin sulfate, *DS* dermatan sulfate, *HA* hyaluronic acid, *HS* heparan sulfate, *KS* keratan sulfate, *N/A* not applicable

^b^When mutations were not determined, the diagnosis of specific MPS type was based on analysis of urinary GAG levels, with indication of kind(s) of GAG(s) with elevated amounts in tested samples, and biochemical determination of deficiency of particular lysosomal enzyme in leukocytes

^c^Catalog numbers are according to cell line description in Coriell Institute

## Results

**Fig. 1:**
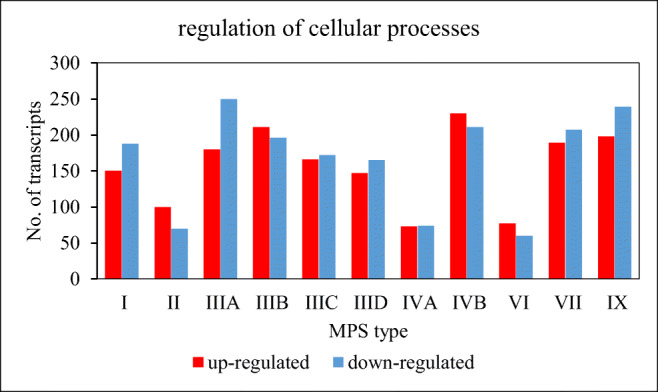
Number of transcripts coding for proteins involved in the regulation of cellular processes with changed levels of expression (at FDR < 0.1; *p* < 0.1) in different types of MPS relative to control cells (HDFa)

Using transcriptomic data obtained from fibroblasts derived from patients suffering from all types and subtypes of MPS, as well as control fibroblasts, we have analyzed transcripts with significantly changed levels in MPS relative to the control, derived from genes listed in the Ensembl database in the term ‘regulation of cellular process’ (GO:0050794), according to the Gene Ontology Consortium. We found that there were hundred or more up- and down-expressed transcripts coding for proteins involved in the regulation of cellular processes in each MPS type/subtype (Fig. 1). The changes appeared more abundant in MPS types I, III (all subtypes), IVB, VII and IX than in types II, IVA and VI. Numbers of up- and down-regulated transcripts were roughly equal in every types/subtype. These results suggest that regulation of cellular processes can be significantly changed in all types/subtypes of MPS, indeed.

**Fig. 2:**
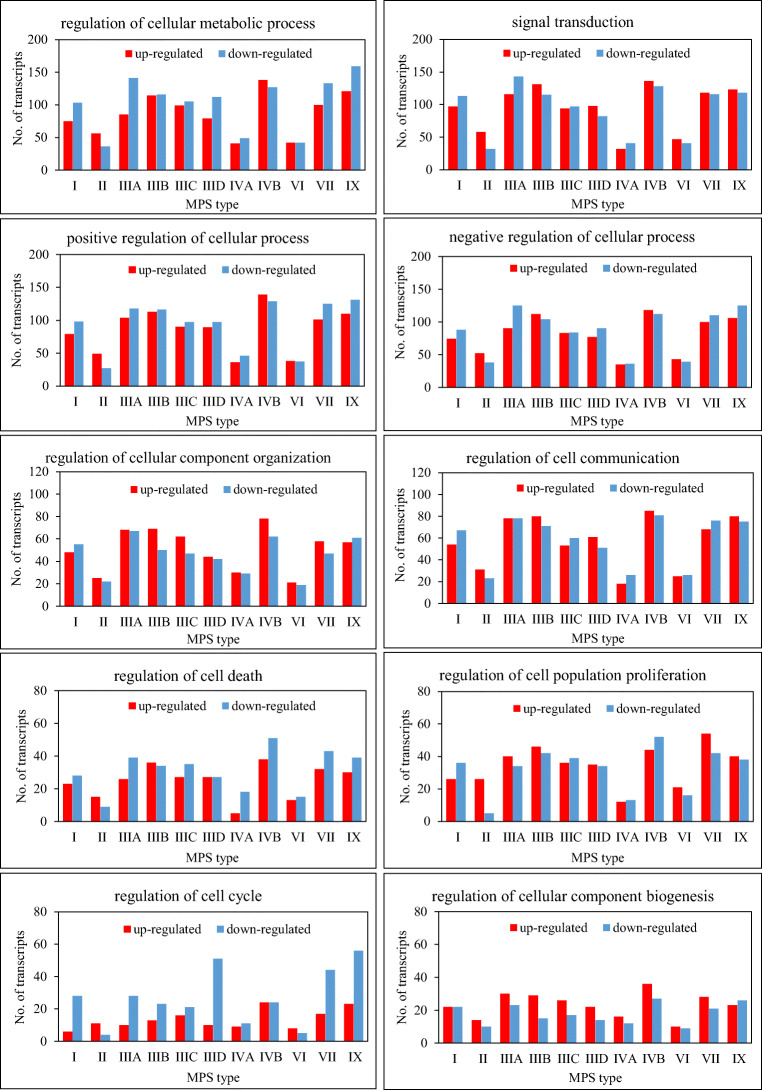
Number of transcripts, corresponding to genes from indicated sub-processes (child processes) of GO:0050794 (regulation of cellular process), defined according to the QuickGO database, which levels were significantly changed in MPS cells relative to the control cells

In the next step, we have analyzed direct children terms of GO:0050794 (regulation of cellular process). Again, as depicted in Fig. 2, significant numbers of transcripts with considerably changed levels have been noted in all MPS types/subtypes when considering following GO terms: regulation of cellular metabolic process (GO:0031323), signal transduction (GO:0007165), positive regulation of cellular process (GO:0048522), negative regulation of cellular process (GO:0048523), regulation of cellular component organization (GO:0051128), regulation of cell communication (GO:0010646), regulation of cell death (GO:0010941), regulation of cell population proliferation (GO:0042127), regulation of cell cycle (GO:0051726), regulation of cellular component biogenesis (GO:0044087).

**Fig. 3:**
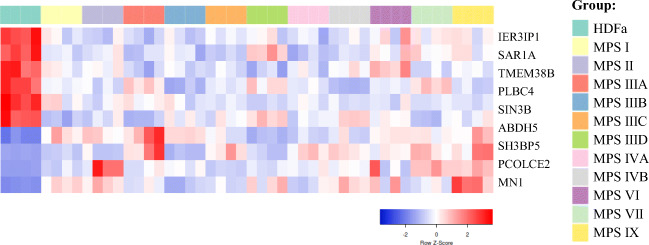
Heat map of transcripts coding for proteins involved in regulation of cellular processes which expression was significantly changed in at least nine MPS types/subtypes relative to the control cells

When assessing genes whose expression is significantly changed in most MPS types/subtypes, we have found that 10 genes are up- or down-regulated in at least 9 MPS types/subtypes (Table 2). These genes include *IER3IP1, SAR1A, TMEM38B, PLCB4, SIN3B, ABHD5, SH3BP5, CAPG, PCOLCE2,* and *MN1*. The first six genes from this group were down-regulated in all MPS types/subtypes while the last four genes were up-regulated in all MPS types/subtypes, indicating that there is a common pattern of expression dysregulation of these genes. Heat map representing these changes is shown in Fig. 3.

**Table 2:**
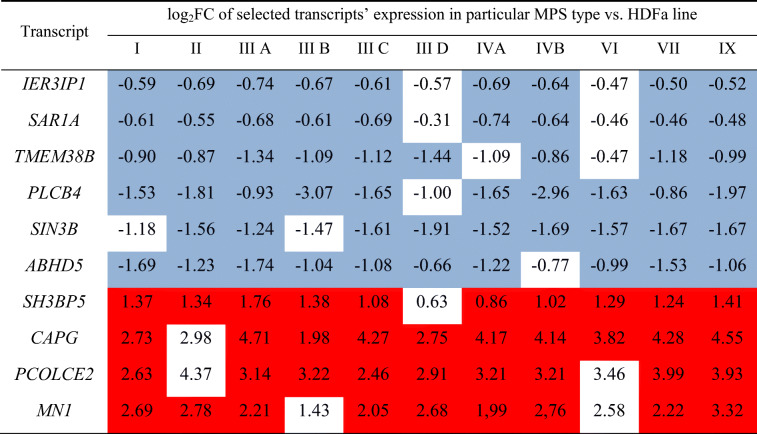
Genes coding for proteins involved in regulation of cellular processes which expression was significantly changed in at least nine MPS types/subtypes relative to the control cells, with indication of log_2_FC

Colored boxes indicate statistically significant differences (at FDR < 0.1; *p* < 0.1) between MPS and control cell lines; blue boxes indicate down-regulation, and red boxes indicate up-regulation relative to control

**Fig. 4:**
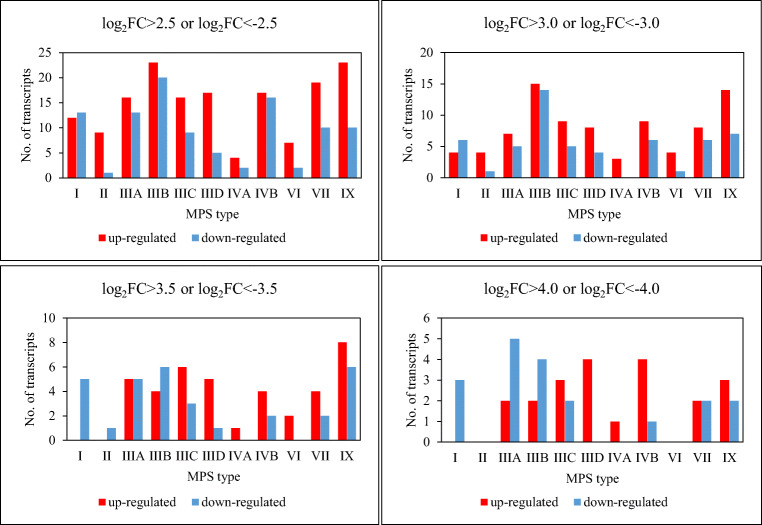
Number of transcripts coding for proteins involved in regulation of cellular processes with particularly high changes in levels in MPS fibroblasts relative to control cells

We have also analyzed genes whose expressions were especially highly changed in MPS cells relative to control fibroblasts. Thus, we assessed transcripts with log_2_ of fold change (FC) exceeding 2.5, 3.0, 3.5, 4.0. Numbers of such transcripts were significant in each of these groups (Fig. 4). The list of genes in which log_2_FC exceeds 4.0 in any MPS type/subtype includes: *WISP2, RARRES2, APOE, TNFRSF11B, MMP3, CXCL8, PTGS1, WISP2, CAV1, SNX3, MMP12, CD9, COMP, TFPI2, IGFBP5, CAPG, OXTR, KRT19, CRLF1, CRIP1*, and *NME2* (Table 3).

**Table 3:** Genes in which log_2_FC exceeds 4.0 in any MPS type/subtype vs. control cell line

Transcript	Especially significantly changed expression (log_2_FC > 4.0 or log_2_FC < −4.0) in particular MPS type vs. HDFa line										
	I	II	III A	III B	III C	III D	IVA	IVB	VI	VII	IX
*WISP2*	–	–	↓	↓	–	–	–	–	–	–	–
*RARRES2*	–	–	↓	↓	–	–	–	–	–	–	–
*APOE*	–	–	↓	–	–	–	–	–	–	–	–
*TNFRSF11B*	–	–	–	–	–	–	–	–	–	↓	–
*MMP3*	–	–	–	–	–	–	–	–	–	–	↓
*CXCL8*	↓	–	↓	–	–	–	–	–	–	↓	–
*PTGS1*	↓	–	–	–	–	–	–	–	–	↓	–
*WISP2*	–	–	–	↓	–	–	–	–	–	–	–
*CAV1*	↓	–	–	–	–	–	–	↓	–	–	–
*SNX3*	–	–	–	–	↓	–	–	–	–	–	–
*MMP12*	–	–	↓	↓	↓	–	–	–	–	–	↓
*CD9*	–	–	–	↑	–	–	–	↑	–	–	–
*COMP*	–	–	–	–	–	↑	–	↑	–	–	–
*TFPI2*	–	–	–	–	–	–	–	–	–	↑	–
*IGFBP5*	–	–	–	–	–	↑	–	–	–	–	↑
*CAPG*	–	–	↑	–	↑	–	↑	↑	–	↑	↑
*OXTR*	–	–	–	↑	↑	↑	–	↑	–	–	↑
*KRT19*	–	–	–	–	–	–	–	↑	–	–	–
*CRLF1*	–	–	–	–	–	↑	–	–	–	–	–
*CRIP1*	–	–	↑	–	–	–	–	–	–	–	–
*NME2*	–	–	–	–	↑	–	–	–	–	–	–

Down-regulated genes are marked with down-headed arrows, and up-regulated genes are marked by up-headed arrows

## Discussion

Recent studies clearly indicated that secondary and tertiary changes (after the primary GAG storage) in cellular processes contribute significantly to the development of disorders and symptoms appearing in the course of MPS (Gaffke et al. 2019; Fecarotta et al. 2020). Significance of this aspect of MPS pathomechanism has been highlighted recently by discoveries of multiple changes in expression of genes coding for proteins involved in various cellular mechanisms in MPS (Brokowska et al. 2020; Gaffke et al. 2020; Pierzynowska et al. 2020; Rintz et al. 2020). Therefore, in this work, we have analyzed transcriptomic data, focusing on expression of genes coding for regulatory proteins responsible for the control of cellular processes. We have used transcriptomic data based on analysis of biological samples derived from patients suffering from all types and subtypes of MPS. This allowed us to obtain a global picture of changes in levels of transcripts in MPS which is an advantage of such an experimental system. The obvious limitations were the use of one cell line from each MPS type/subtype (due to technical reasons), which cannot reflect a variability between patients suffering from the same MPS type, and employment of fibroblasts which represent only one type of cells. Nevertheless, confirmation of transcriptomic results, obtained for selected genes by the RT-qPCR method (reported previously by Gaffke et al. 2020; Pierzynowska et al. 2020; Rintz et al. 2020), as well as the fact that expression of particular genes was generally either up- or down-regulated in all/vast majority of MPS types/subtypes, strongly suggest that these analyses are reliable.

Here, we demonstrated that expression of hundreds of genes coding for regulators of cellular processes is changed in each MPS type/subtype. When assessing genes whose expression is significantly dysregulated, relative to control cells, in at least 9 MPS types/subtypes, following ten were found: *IER3IP1, SAR1A, TMEM38B, PLCB4, SIN3B, ABHD5, SH3BP5, CAPG, PCOLCE2,* and *MN1.*

IER3IP1 is the immediate early response 3 interacting protein 1. This protein is localized to the endoplasmic reticulum (ER), and it was suggested to play a role in the ER stress response. Possibly, this function can influence cell differentiation and apoptosis. Mutations in the *IER3IP1* gene were connected to severe dysfunctions in CNS, and the symptoms include epilepsy (Valenzuela et al. 2017). Expression of *IER3IP1* is down-regulated in all MPS types/subtypes, thus, one might suggest that lower levels of the gene product may contribute to neurological symptoms in MPS patients.

The *SAR1A* gene encodes secretion associated Ras-related GTPase 1A. This protein is involved in the transport from ER to the Golgi apparatus (Donaldson and Jackson 2011). Since *SAR1A* expression is impaired in all MPS types/subtypes, it is likely that SAR1A deficiency may partially cause the problems with vesicular transport, reported for MPS (for a review, see Gaffke et al. 2019).

The maintenance of intracellular calcium release is partially dependent on the function of the transmembrane protein 38B, a monovalent cation channel, encoded by the *TMEM38B* gene. Mutations in *TMEM38B* were connected to osteogenesis imperfecta (Valadares et al. 2014), and skeletal problems are common in MPS. Thus, decreased levels of *TMEM38B* transcripts in MPS cells may be partially responsible for such symptoms.

*PLCB4* encodes phospholipase C β4. Formation of inositol 1,4,5-trisphosphate and diacylglycerol from phosphatidylinositol 4,5-bisphosphate is catalyzed by this enzyme. Therefore, decreased expression of *PLCB4*, observed in MPS, may impair intracellular transduction of many extracellular signals. In fact, dysregulation of *PLCB4* signaling has been suggested to be linked to various brain disorders (Yang et al. 2016) which may indicate contribution of phospholipase C β 4 deficiency to neurodegeneration in MPS.

Transcription regulator family member B, encoded by the *SIN3B* gene, is involved in the regulation of cell cycle progression (Kadamb et al. 2013). Thus, significantly changed expression of this gene in cells of most MPS types/subtypes indicates that cell proliferation disturbance in MPS cell may partially result from improper levels of this transcription repressor.

The *ABHD5* gene codes for one of abhydrolases. Mutations in *ABHD5* are associated with a triglyceride storage disease (Missaglia et al. 2019). Therefore, one might presume that decreased expression of this gene in MPS may contribute to secondary storage, often observed in this disease.

The SH3 domain binding protein 5, encoded by *SH3BP5,* is a negative regulator of the phosphorylation activity of BTK, one of Tec family kinases (Ortutay et al. 2008). The *SH3BP5* gene product may be involved in the control of apoptosis, thus, one can propose that it contributes to dysregulation of this process, reported in MPS (Brokowska et al. 2020).

The *CAPG* gene codes for capping actin protein, gelsolin like (Nag et al. 2013), contributing to the control of actin-based motility. Dysregulation of expression of this gelsolin-like protein may result in functional problems of actin cytoskeleton in MPS.

*PCOLCE2* encodes procollagen C-endopeptidase enhancer 2 (Sorci-Thomas et al. 2015). Its enhanced expression in MPS cells may indicate disturbed regulation of collagen metabolism.

The *MN1* gene encodes a transcription factor which may be involved in development of meningioma (Handschuh 2019). Therefore, enhanced expression of *MN1* in MPS cells might potentially cause various defects in CNS.

The above analysis indicates that significant changes in expression of genes coding for various regulators of cellular processes may considerably contribute to the development of cellular dysfunctions, and further appearance of specific symptoms of MPS, including neurodegeneration. This conclusion may be supported by the analysis of genes whose expression is particularly severely dysregulated in some MPS types/subtypes. The list of such genes includes *APOE* (coding for apolipoprotein E)*, OXTR* (coding for oxytocin receptor) and other genes coding for proteins involved in the control of crucial cellular processes. Further studies on molecular mechanisms of cellular changes related to disturbed expression of various genes should indicate specific processes which are dysregulated in MSP cells, leading to better understanding of pathomechanisms in this group of diseases.

In summary, transcriptomic analyses presented in this work not only indicated genes coding for proteins involved in regulation of cellular processes whose expression is significantly changed in fibroblasts of different MPS types (Fig. 1), including those affected in most types (Table 2, Fig. 3), but also suggested what processes may be especially dysregulated. These include metabolic processes, signal transduction, organization of cellular components, cell communication, cell death, cell population proliferation, cell cycle, and biogenesis of cellular components (Fig. 2). Interestingly, the genes with most severely changed expression were either up- or down-regulated in all or vast majority of MPS types (Table 3, Fig. 4), suggesting that there are groups of processes similarly changed in all these types, despite evident specificity of each type. Although experiments presented in this report were performed on fibroblasts, specific changes in expressions of regulatory proteins might, to some extent, be extrapolated to other cell types, including neurons as discussed previously (Pierzynowska et al. 2020), suggesting that they could contribute to neurodegenerative processes occurring in MPS.

## Data Availability

RNA-seq data, deposited in the NCBI Sequence Read Archive (SRA), are available under accession number PRJNA562649.
